# Rasterstereographic measurement of scoliotic deformity

**DOI:** 10.1186/s13013-014-0022-7

**Published:** 2014-12-12

**Authors:** Burkhard Drerup

**Affiliations:** Bundesfachschule für Orthopaedietechnik, Schliepstrasse 6-8, Dortmund, Germany

**Keywords:** Back surface, Scoliosis, Rasterstereography, Photogrammetry, Curvature map, Symmetry line, Anatomical landmarks, Shape analysis, Cobb angle

## Abstract

**Background:**

Back surface topography has gained acceptance in recent decades. At the same time, the motivation to use this technique has increased. From the view of the patient, the cosmetic aspect has played and still plays a major role as it provides a comprehensive documentation of cosmetic impairment. From the view of the medical practitioner, the aspect of reducing X-ray exposures in diagnosis and follow-up has been dominant and still prevails. Meanwhile, new aspects have emerged: due to the consequent three-dimensional view of the scoliotic condition, treatment success can be visualized convincingly. Clinical diagnosis is supported by information otherwise not supplied by X-rays, such as when functional examinations and diagnostic tests are recorded.

**Methods:**

Like rasterstereography, most techniques of actual back surface measurement refer to photogrammetry and the triangulation method. However, with respect to the particular clinical application, a wide spectrum of implementations exists. Applications in a clinic require high accuracy of measurement in a short time and comprehensive analysis providing data to be used to supplement and compare with radiographic data. This is exemplified by rasterstereography; the procedures of surface analysis and localization of landmarks using curvatures and the reconstruction of the spinal midline will be described.

**Orthopaedic relevance:**

Based on rasterstereographic analysis, different geometrical measures that characterize the back surface are given and underlying skeletal structures described. Furthermore, in analogy to radiological projection, a 3-D reconstruction of the spinal midline is visualized by a frontal and lateral projection, allowing comparison with pertinent X-rays.

**Conclusions:**

Surface topography and, in particular, rasterstereography provide reliable and consistent results that may be used to reduce X-ray exposure. Unfortunately, the correlation of shape parameters with the radiological Cobb angle is poor. However, the wealth of additional applications substantially enhances the spectrum of clinical value.

## Introduction: measurement of spinal shape

### Motivation

Scoliosis and other deformities of the trunk and spine not only have an adverse effect on the physiological function but also provoke a severe impairment of the outer appearance of the patient. Therefore, the first contact of afflicted children and adolescents in consultation with a medical professional is often due to cosmetic reasons. The medical diagnosis then is based on a clinical examination and on X-rays. In case a spinal deformity is evident, follow-up X-ray examinations at regular time intervals are inevitable, burdened with a radiological hazard [1].

There are several options to reduce the radiation risk, for example, by prolonging the intervals of X-ray examination and bridging these intervals with alternative examinations. Those examinations, however, must follow the severity of the condition and must be capable of indicating when a further X-ray examination is necessary. This usage at present is one of the strongest motivations to apply optical surface measurement. Indeed, in recent years, back surface measurement has proved to be in part suitable for this purpose [2-4]. However, the amount of effort it entails could not be estimated or even foreseen during the initial period around 1980. Instead, the appeal of reducing radiation prevailed and thus boosted the development of surface topography. This primary motivation even eclipsed other motivations that were truly in favour of back surface measurement as well, as we know today. Particularly worth mentioning from today’s perspective is the ease of repeating optical recordings, thus opening up possibilities for functional examinations. Another strong argument is that it brings together skeletal and surface information, allowing for a deeper understanding of biomechanics and pathogenesis, as was urged by Burwell and co-workers [5], and last but not least, it helps to document cosmetic aspects in three dimensions and is convincingly intelligible to the patient as well.

### Early expectations

The present appreciation of back surface measurement differs from the initial expectations placed on the new technology between 1970 and 1990. At the early stage, well-established clinical methods in assessing trunk asymmetry were hoped to provide evidence of the spinal curve if applied with sufficient accuracy, such as the methods of Burwell [5] or rib hump indexing [6] or, in a simpler version the skoliometer [7]. That accuracy required these techniques to be applied objectively, more frequently, more precisely and almost simultaneously at different points on the back. In parallel, new technologies boosted these expectations when enhanced digital computer power and new video techniques allowed for fast and cheap methods of surface recording. Paradoxically enough, however, this development started in 1970 with a publication on moiré topography [8], not a truly digital and initially not a video technique but instead an analogue photographic technique. It should be mentioned for completeness that the publication of Takasaki [8] occurred 2 months after publication in the same physics journal of rather the same method [9] but that the earlier article visualized a technical application instead of a cosmetic and potentially medical application. The cosmetic/medical application was demonstrated by Takasaki, and the merits of introducing moiré topography in this field have been attributed to him since then. Using the moiré method produces a so-called topogram of the back, which exhibits contour lines overlaid on a photograph of the back. Thus, the advantages of a photograph with the addition of a depth map are combined. As a result, appealing representations are produced, supporting the imagination to provide easy access to the complexity of 3-D data. It is therefore reasonable that this attempt has fascinated the scientific community dedicated to the diagnosis and treatment of scoliosis [10].

In this context, some orthopaedic researchers initially may have looked at the contour lines as some sort of fingerprint of the deformed spine that might be interpreted immediately, e.g., by visual inspection without assistive devices, but nevertheless allowed for the provision of measures like the Cobb angle and the localization of apices. However, those expectations failed, and soon the interest was directed to conventional orthopaedic measurements, indexes and grades that traditionally were taken with rulers and goniometers. The almost euphoric hope was that the improved quality and quantity of this type of data should provide more reliable information on spinal shape, and thus radiographs might be replaced at least in part. It was a long process to realize that despite continuous improvement of the methods of image recording and of enhanced computer power for image processing, the success of these methods was limited by other factors. Again, this process was expedited in the beginning by scrutinizing moiré topograms [11,12] as the topograms clearly revealed the contour line patterns to be highly variable and to depend not only on variations of posture but on variations of positioning as well. Here positioning means position relative to the measurement device. In both cases, even small variations may produce striking effects on the contour line pattern and thus on the perception of the back shape. While uncertainties in positioning may be resolved by padding and appropriate positioning devices, postural uncertainties remain and even may be intensified as by-products when positioning devices are applied. Finally, this confusing situation supported the insight that there is not a common one-to-one relation between back shape and spinal shape but that instead a complex linkage exists between these two entities.

### The fuzzy interrelations of back shape and spinal shape

In the case of a physiologic spine, the spinal curve is restricted to the sagittal plane, exhibiting a sequence of kyphotic and lordotic curves. Therefore, the relation between spinal shape and the back profile is limited to two dimensions. It is evident that an interrelation must exist between the spine and back curves, thus allowing a conclusion from the back surface contour to the spine. However, the link between the two curves is established by the spinous processes, which may exhibit individual variations in length and inclination against the vertebral endplates; thus angular discrepancies are introduced. While the Cobb angle refers to the vertebral endplates – more precisely, their projections – the back contour refers to the line of spinous processes. Therefore, in predicting the Cobb angle from the outer contour [13-15], considerable uncertainties may be induced. That is, at least, the case in an initial comparison of an X-ray and the surface profile of the same patient. However, it may be assumed that possible changes of the X-ray curve are paralleled by the surface curve due to their strong interrelation. Therefore, back surface measurements in kyphosis and lordosis often are confined to follow-up examinations looking for changes in these curves instead for the absolute values.

In the case of scoliosis, the situation is far more intricate. Due to the three-dimensional character of the condition, the spine exhibits a combination of translations and rotations in all planes. In the sagittal plane the kyphotic curve is no longer harmonic. Instead it exhibits in adjacent zones local hyperkyphotic and hypokyphotic curves. Furthermore the component of lateral deviation of the vertebrae is difficult to observe on its own. It becomes apparent only because of a complex chaining of biomechanical causes and effects. On the one side they combine motions of vertebral rotation and lateral tilt. On the other side, they create biomechanical implications for the rib cage and scapulae, effectuating a 3-D deformation of the trunk. In an attempt to reverse these relations, different approaches for the conclusion from the back surface to the spine have been proposed. One starts from back shape features to perform some classifications of the trunk deformity. It may provide, on a statistical basis, an estimation for the type and severity of the spinal deformation [16,17]. Another attempt [18] considers the deformities of the spine and of the trunk as two distinct components of scoliosis deformity. Here, too, no biomechanical modelling is applied. In both attempts, a relation is postulated, but a considerably random component remains. As a third attempt, the rasterstereographic reconstruction of the spine will be described later.

### Requirements into back shape measurement and spinal reconstruction

Neugebauer [19] described what was needed from his view: “A new method, which is practicable, repeatable and sufficiently precise – but without radiation exposure”. For this it is a prerequisite measurement of the back surface must be performed with high accuracy. This requirement is met best with photogrammetric techniques. Nevertheless, given that and supposing a reliable reconstruction of the back surface is provided, this is not yet sufficient for applications in scoliosis. This is partly due to the particularities of back shape that are in contrast to, e.g., technical surfaces with predefined basic geometrical elements. The back shape is not completely determined by a few geometrical parameters but requires a more elaborate approach considering the back surface to be irregular and variable. In addition, a simple and one-to-one relation between back surface and spinal shape does not exist, as has been described above. This circumstance necessitates, first, an advanced mathematical and geometrical analysis to characterize the shape and, then, a separate step to establish analytically a model of the deformed spine based on anatomical findings and biomechanical principles. Finally, a detailed valuation must be enabled and provided. That means that a quantitative comparison of the 3-D reconstruction with radiological and clinical findings is possible. Therefore, the spine must be provided in a way that is compatible with standard X-rays – i.e., in a frontal or lateral projection. Beyond that, parameters characterizing the geometric configuration of selected points on the back surface and on the skeleton must be provided. They are used as an interface for clinical measures and to support a better understanding of the deformity.

To achieve a reliable reconstruction of the spinal curve it turned out that a thorough understanding of the underlying biomechanics of the spine and the interrelations between back shape and spinal shape are inevitable. The biomechanical understanding of spine mechanics needed for the reconstruction of the spine focuses on the behavior of the whole spine. This may be realized if characteristics like lateral deviation and vertebral rotation are modelled by functions of spinal level which in addition are closely related [20].

An example of the realization of this concept is the method of rasterstereographic back shape measurement. This concept will be outlined in the following. The methodological section describes the photogrammetric concept of rasterstereography providing a 3-D model reconstruction of the back. To allow application in scoliosis management, other aspects must be added: Mathematical methods must be provided to establish the recognition of peculiar shapes, particularly the identification of anatomical landmarks. Furthermore, an algorithm must exist to establish the symmetry line as a prerequisite in the reconstruction of the spinal midline.

As the term “rasterstereography” is increasingly used as an umbrella term for various measurement methods projecting a regular pattern of lines to the back, it is noted here that in the following, this term refers to the original method of rasterstereography [11,21,22]. In this method, one single exposure of the back with a projected line pattern is recorded. It is followed by a photogrammetric evaluation providing input data for a biomechanical evaluation.

## Methods

### Photogrammetry

Using the term “measurement of back surface” illustrates that the back is recorded as a whole instead of as a small number of points that have been marked prior to the measurement. This implies that a large number of points distributed over the whole back is taken, each one with high accuracy in all three dimensions. In addition to that, the measurement points should exhibit a regular distribution on the surface to allow for advanced methods of mathematical surface analysis.

**Figure 1:**
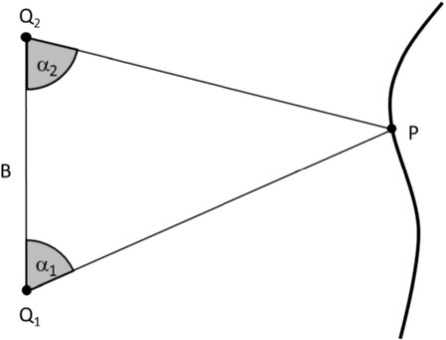
**Schematic of the principle of triangulation.** In the plane of the drawing, the point P on an object is uniquely defined if the base B with the two points Q_1_ and Q_2_ and the two angles α_1_ and α_2_ are known.

The technical solution in recording a surface point with high accuracy is based on the methods of photogrammetry, which in turn are based on the geometrical method of triangulation. According to this method, the 2-dimensional coordinates of a point P can be measured, if the so called stereo base made up by two points Q_1_ and Q_2_ is known and the angles α_1_ and α_2_ (Figure 1) are measured. To achieve the 3-dimensional coordinates, the same stereo base may be used, but in Q_1_ or Q_2_ a second angle has to be measured in a plane perpendicular to the plane for measuring P in 2 dimensions.

**Figure 2:**
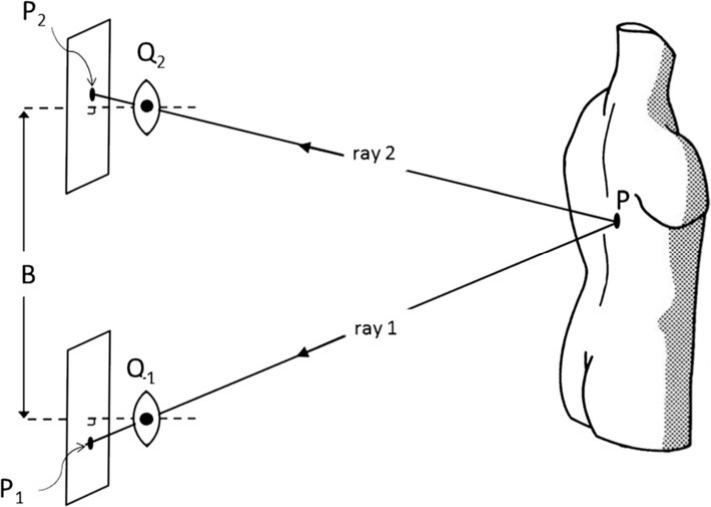
**Principle of stereophotogrammetric setup.** The base B now is defined by the lenses of two cameras that have been placed into the points Q_1_ and Q_2_. The angles are measured from the coordinates of the image points P_1_ and P_2_.

Triangulation is applied in a wide range of modifications. Often, the angles α_1_ and α_2_ are determined photographically or by video techniques. For that purpose, two cameras are used, with the photographic lenses placed into the points Q_1_ and Q_2_. The angles then are measured by capturing in the film plane the position of the image point P_1_ relative to Q_1_ and of P_2_ relative to Q_2_ (Figure 2). For use in medical applications, it has proved to be an indispensable requirement that recording and reconstruction of the back surface be processed automatically. This feature is largely facilitated if one camera is replaced by a projector, with the advantage that then only one image must be recorded for a measurement. The second image, which is necessary for triangulation, is provided by a slide that has been measured once and for all as an extension of the calibration process. Using a projector together with a camera is today a general characteristic of all actual systems [23].

As a prerequisite for triangulation, the stereo base must be determined with high accuracy in a separate process called calibration. It typically comprises the determination of the position of the imaging system in space and furthermore the determination of all relevant geometrical parameters, for example, the focal length of the camera and the projector [23].

As far as the principle of triangulation is concerned, quite different methods like moiré topography [8], Isis [24,25], coded light approach [26] and rasterstereography [27] apply the same measurement principle and therefore may achieve the same accuracy. Nevertheless, some distinctions remain. They are due to the particularities of the realization, for example, the time needed for the measurement. It is obvious that sequential scanning with a light section [24] will take a longer time than recording the whole surface with only one video frame, and thus inaccuracies or blurring caused by movement may occur. Other particularities with consequences for the accuracy and density of measurement points pertain to the light pattern projected onto the back and the density of measurement points [23]. Depending on the application, different solutions have been realized. Nevertheless, the projection of parallel line patterns has widely been established.

### Rasterstereographic image capture and reconstruction

**Figure 3:**
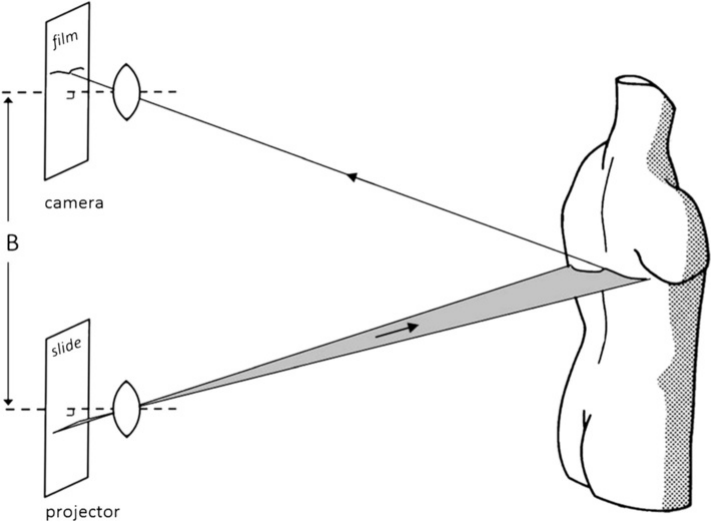
**Principle of rasterstereophotogrammetry containing a projector and camera.** One single light section is projected onto the back. The image of the deformed section is recorded in the film plane.

An extensive discussion of the photogrammetric principles realized in rasterstereography is given by Frobin and Hierholzer [21,27]. In the following section, only some key features will be described. The underlying method may be described in technical terms as a simultaneous multi-light-sectioning procedure. It is a light-sectioning method because a light plane produced by a slide is projected onto the back (Figure 3). The deformation of the projected line seen in the camera – in combination with the calibration data – allows for 3-D reconstruction of the sectioned surface points by methods of triangulation. The term “multi-light sectioning” indicates that several, in total 81, light sections are projected, covering the whole back by a regular system of lines. Furthermore, it is a simultaneous procedure as all lines are projected simultaneously and are recorded with a video camera in one single frame taking 1/25 sec only. This feature largely prevents blurring by patient movements. In addition, the study of motions is enabled by capturing a series of frames in a dense time sequence.

**Figure 4:**
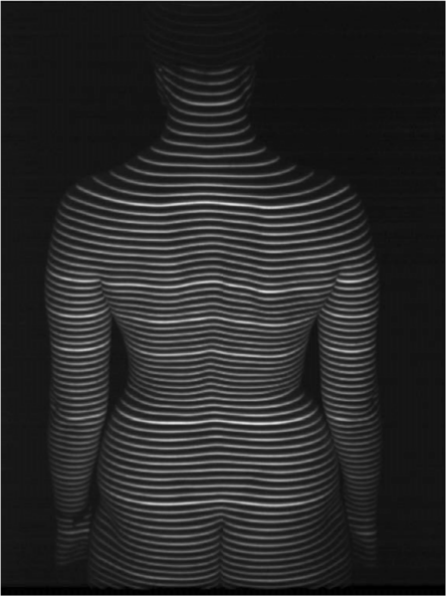
**Projected line pattern for videorasterstereography in the perspective of the camera (from** **[** 28 **]** **).**

Figure 4 shows a typical single frame image of the back that provides the input data for the reconstruction of the model of the back and the subsequent mathematical analysis of the back shape. The scheme of thick and thin lines is used to unambiguously identify the light sections. A unique identification of the lines is a prerequisite for the photogrammetric reconstruction. Thus 3-D information can be supplied by evaluating the camera image only.

**Figure 5:**
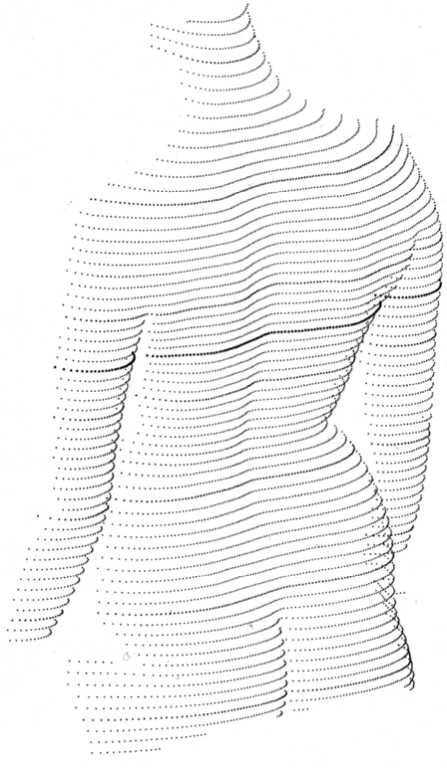
**Perspective view of the reconstructed back (from** **[** 28 **]** **).**

Regarding the density of measurement points, the light-sectioning method provides a dense sequence of points along each projected line. However, only surface points hit by a line can be measured. In reverse, surface structures falling totally into the gap between adjacent lines are not measured. The gap between adjacent lines on the back in a typical measurement situation is about 11 mm. Structures like vertebra prominens or the lumbar dimples (often denoted as “dimples of Venus”) are typically hit by two, three or even four lines. Therefore, the line density is sufficient [29,30]. A further increase in line density does not appear to be advantageous as practical limits arise; for example, light scattering in the skin leads to a blurring of the lines. For a medium-sized subject, typically 25000 primary measurement points are recorded. They are reduced for purposes of smoothing and data reduction by interpolation. As a result, a homogeneous distribution of typically 8000 points is obtained, each with a depth resolution of typically 0.2 mm. The interpolated points are arranged in regular distances along a regular scheme of horizontal sections. Figure 5 shows in an oblique perspective the reconstruction of the back recorded in Figure 4. The scheme of thick and thin lines has been applied only for better visual identification of the sections. They are independent of the thick and thin lines projected on the back in Figure 4.

### Rasterstereographic shape analysis

As a result of the model reconstruction, the back surface initially is given as a set of 3-D surface points in a computer file only. This may be looked at as equivalent to a replica of the back that might be produced as a plaster cast or by use of a 3-D printer. However, the real task still remaining is to interpret the shape, i.e., to extract relevant parameters from the surface data characterizing and quantifying the back surface.

The underlying motivation may become clear when compared with a blindfolded clinical investigator examining the model of the back with his fingertips to localize anatomical landmarks. While these structures may be hard to detect by visual inspection only, they are revealed to the fingertips due to their peculiar shape, which is characterized by a unique curvature of the surface in the landmarks and in their neighbourhood. The fingertips will localize the landmarks irrespective of whether the model is standing or lying in front of the investigator. Therefore, the term “shape” is used here to describe relevant information on a geometrical configuration independent of its location and orientation.

**Figure 6:**
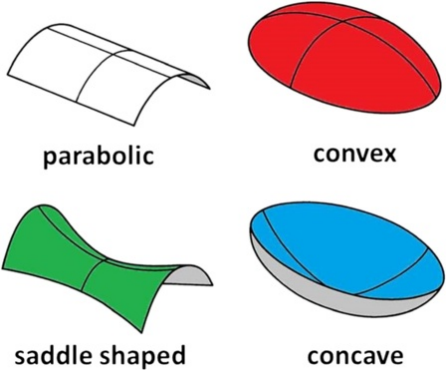
**Basic types of surface curvature.** Colours refer to curvature maps Figures 7 and 8.

In performing an analytical examination of the surface using mathematical methods, the concept of surface curvatures has proved to be extremely helpful [31,32]. The particular procedures are embedded in the mathematical discipline of differential geometry. Due to the focus put here on the analysis of curvatures, this kind of analysis is denominated as *curvature analysis*. According to this concept, the shape of a small surface patch may be characterized by the magnitude of the pertaining curvature and by distinguishing four principal types of curvature, namely parabolic, convex, concave and saddle-shaped curvature. In the graphical representation in Figure 6, they are given the colours white, red, blue and green respectively.

**Figure 7:**
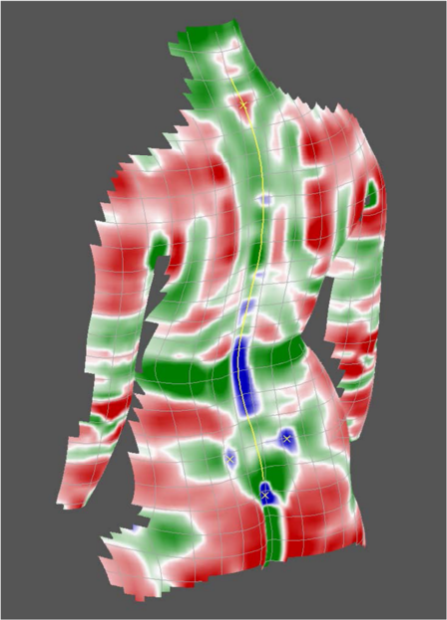
**Map of Gaussian curvatures.** The representation discriminates between saddle shaped, concave and convex regions (from [28]).

**Figure 8:**
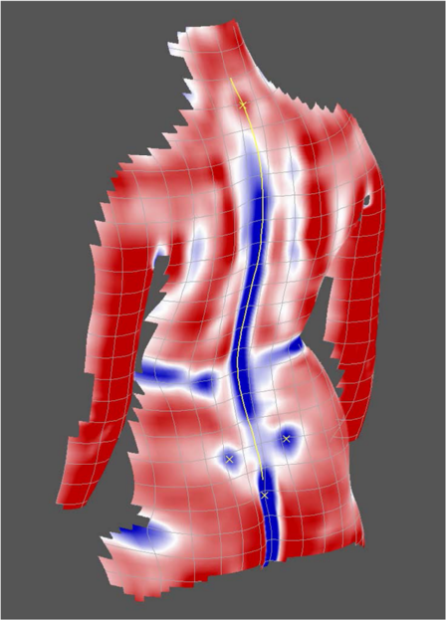
**Map of mean curvature (from** **[** 28 **]** **).**

Figure 7 shows a so-called *curvature map* displaying curvatures detected on a reconstructed model of the back. Using different colours, it discriminates convex, concave and saddle-shaped regions of the back while the intensity of colouring indicates the magnitude of curvature. The transition regions between the different types of curvature are white. They thus indicate their parabolic character, meaning that these patches may be flattened onto a plane without distortion. An alternative representation of the same back is obtained in the curvature map of Figure 8, which discriminates between convex and concave shapes only, again with parabolic regions of transition. Saddle-shaped regions are not explicitly displayed. They are indicated as convex or concave regions depending on the dominant curvature. In terms of differential geometry, this type of representation is denominated as *mean* curvature. The alternative algorithm discriminating also saddle-shaped curvatures is denoted as *Gaussian* curvature.

### Anatomical landmarks

The two curvature maps in Figures 7 and 8 reveal the practical significance of surface curvatures. Anatomical landmarks [33] typically are characterized by their particular shape, and they therefore provide a specific pattern of curvatures. In the curvature maps, they are indicated by yellow x-marks.

In the Gaussian curvature map, the vertebra prominens landmark, for example, exhibits an isolated red – i.e., convex – region surrounded by a green – i.e., saddle-shaped – region. The exact localization of this point on the surface is defined by the point of maximum convex curvature. To verify that this point coincides with the tip of the spinous process, studies have been performed where it has been palpated and X-rayed. In both cases, the coincidence with the maximum of curvature was found to be in the range of a few mm [34,35]. Interpretation in terms of anatomy localizes the vertebra prominens most frequently at C7 [36] but as it has been pointed out [37], it might mark the tip of T1 as well.

The lumbar dimples provide two other anatomical landmarks. The Gaussian curvature map reveals the dimples typically as blue – i.e., concave – regions. In some cases, the dimples are detected more clearly in the mean-curvature map. Thus, the mathematical algorithm for their localization must consider both representations. The dimples establish a link to the pelvis, or more precisely, to the posterior superior iliac spines (PSIS). However, in contrast to the vertebra prominens landmark, they are not situated exactly over the corresponding bony landmarks but are shifted by 5 to 10 mm in a lateral and cranial direction from the bony landmarks [38] with a good individual reproducibility. Further improvements of the localization algorithm have been proposed [39].

**Figure 9:**
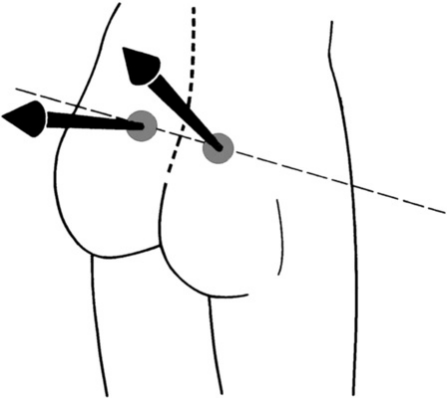
**Anatomical landmarks of the dimples.** The arrows symbolize the surface normals providing information on the orientation and torsion of the pelvis.

In addition to their localization, the dimple landmarks are analysed also for the orientation of the surface normals directing perpendicular to the skin on the landmarks (Figure 9). They thus provide relevant measures for the orientation of the pelvis [40]. Other anatomical landmarks of minor relevance in the examination of scoliotic deformity are the sacrum point, i.e., the beginning of the anal cleft (concave), and the tips of the scapulae (convex).

Besides the anatomical landmarks detected as singular points, the so-called symmetry line of the back also can be extracted from the curvature maps [41]. It is indicated as a yellow line in Figures 7 and 8. Mathematically, it is computed as the line dividing the back into two halves with minimal left-right asymmetry. In the symmetric back, the symmetry line runs straight and is completely embedded in the sagittal plane. The same holds for the line of spinous processes. For symmetry reasons, it is bound to the sagittal plane as well, and thus it coincides with the symmetry line on the back. Therefore, in the symmetric back, the symmetry line may be taken as a predictor of the spinous process line. This concept is transferred to the analysis of the asymmetric scoliotic back. In the scoliotic back, a symmetry line can be calculated as well, but it is no longer bound to the sagittal plane. In practice, the resulting symmetry line does not always provide a sufficiently clear and unambiguous solution. Sometimes, different curves provide similar values of asymmetry summed over all spinal levels. Therefore, supplementary restrictions have to be enforced. They smooth the symmetry line and effectuate accordance with biomechanical principles, in particular, the coupling of movements between vertebrae and the behavior of the whole spine with regard to lateral deviation and vertebral rotation [20,42]. In this way, discontinuities and kinks of the reconstructed symmetry line are avoided. Further attempts to improve the symmetry line have been described [43].

In Figures 7 and 8 the symmetry line is depicted in yellow. It extends from a level above the vertebra prominens landmark to the rima ani landmark. Both landmarks must lie, for reasons of symmetry, on the symmetry line.

**Figure 10:**
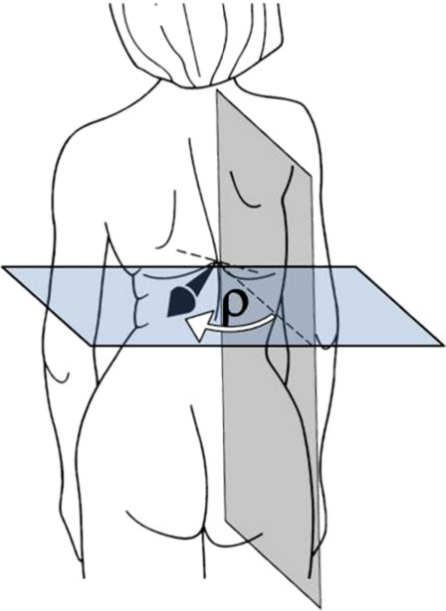
**Schematized scoliotic back with the symmetry line and a horizontal surface normal to the symmetry line.** The angel ρ of surface rotation measures the deviation between the sagittal plane and the surface normal in the horizontal plane.

Another property of the symmetry line is associated with the term *back surface rotation*. In Figure 10 a schematized scoliotic back with the symmetry line and a transverse profile is shown. In the intersecting points, an arrow indicates the direction of the surface normal. The deviation of its direction from the sagittal plane is denoted as the angle ρ of surface rotation.

### Construction of a spine model

In establishing an analytical construction of the midline of the spine, two assumptions about the symmetry line must be made.

1. The symmetry line is a predictor of the line of spinous processes.
2. The surface normals along the symmetry line indicate the vertebral rotation at the particular level.
  According to a proposal of Turner-Smith [24], the reconstruction of the vertebral body line can then be established if two more assumptions are made with respect to the vertebrae:
3. No serious deformations have been caused by the scoliosis condition.
4. The distance L between the centre of a vertebra and the skin covering the spinous process is known as a function of spinal level and of body height of the patient.

**Figure 11:**
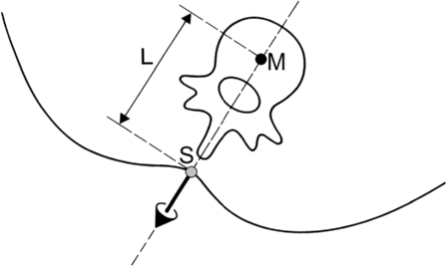
**Construction of the vertebral midpoint in a given horizontal section of the trunk.** Construction of point M by going from surface point S on the symmetry lines by the distance L in opposite direction against normal direction.

The basic idea of the construction algorithm is shown in Figure 11. It shows a transverse section of the trunk. By going a distance L backwards, opposite to the direction of the surface normal from the symmetry line (grey point), the centre point in the vertebral body (black point) is obtained. Connecting the reconstructed points at different levels reveals a 3-D curve, which is taken as a model of the spine in three dimensions. Here it is denoted as the *spinal midline*.

### Positioning devices and surface markers

One option when performing back surface measurement and shape analysis is to minimize the need for assistive devices during measurement. The standing position implies that the patient stands freely in his habitual posture. There is no need for him to activate his muscles to maintain a prescribed posture, and there is no need to use positioning pads to align him perpendicular to the viewing axis of the measuring system. Instead, he is free to exercise some functional test, like forwards or sideways bending.

Nevertheless, the analysis must refer to a suitable coordinate system. In case of rasterstereography, this is accomplished analytically by reference to a so-called *body-fixed coordinate system*. Different coordinate systems may be used. Here it is fixed to the vertebra prominens and the midpoint between the dimples. The sagittal plane is defined by reference to the dimple landmarks. Therefore, the coordinate system is defined by the patient himself; i.e., it moves together with the patient. In this coordinate system, the terms “frontal projection” or “sagittal direction” indicate a direction perpendicular to the plane spanned by the vertebra prominens and the dimple landmarks. All measurement data, surface data, profiles, surface parameters and angles, anatomical landmark data and skeletal data refer to this coordinate system, and therefore an occasional variation in positioning will not affect the results. However, if in addition the posture is varied, this affects the shape of the back surface, and thus all parameters and data characterizing it will change accordingly.

A similar argument applies to using surface markers. They are often attached as adhesive markers at selected points on the skin to indicate specific skeletal structures thereunder or in close neighborhood. These markers then are recorded together with the back surface to establish a link between the surface and the skeletal structures. However, the method is burdened by different sources of error [44,45], like inaccuracies in attaching the markers. As the markers can hardly be localized by visual inspection, a palpating examination is needed for exact localization, which then must be indicated on the skin, possibly leading to errors in the transfer. Another source of error arises from movements of the skin shifting the markers and thus causing false interpretations of their localization. This is not the case if the landmark is localized by shape analysis, which detects the landmarks due to the particularities of surface curvature in their neighborhood. It is evident that these particular curvatures are not fixed to the skin but remain in their position relative to the skeleton, even when the skin is moved. In this way, an objective and reproducible method of linking skeleton and back surface is provided.

### Assessment of reliability

In order to assess the reliability of the reconstruction, a comparison of standard X-rays and rasterstereographic reconstruction has been performed [22]. The comparison comprised 478 pairs of rasterstereographic measurements and corresponding X-rays of scoliotic patients with Cobb angles up to 52°. As a special feature, vertebral rotation measured at each spinal level from X-rays [46] was compared with the pertinent surface rotation. In summary, a mean rms deviation of 3° between vertebral rotation and surface rotation was found. This figure is composed in roughly equal parts by the uncertainties in reconstructing the symmetry line and by different errors in the radiological measurement of vertebral rotation. Thus, the second of the four assumptions in constructing a spine model has been verified on its own.

Another feature investigated for reliability was the lateral deviation. The curve connecting the centres of the vertebral centres in the frontal X-ray was compared with the corresponding projection of the rasterstereographic reconstruction. Summed over all spinal levels, a mean discrepancy between the two frontal projections of 4 mm was obtained [42], indicating a reliable prediction of the spinal deformity from the surface measurement. The result furthermore may be taken as a global verification of the three remaining assumptions in constructing the spine model – at least for scolioses up to 50°. This does not exclude that the conformity may be better or worse depending on factors like the stadium of growth or the severity of the condition including deformations of the vertebrae. Growth for example has been identified [47] to have a significant effect on the correlation between the thoracic and spinal deformity and thus might also affect the correlation between the symmetry line and the resulting rasterstereographic reconstruction and the radiograph. On the other side in severe scolioses exhibiting a Cobb angle of 100° and more, MR tomography has been used to assess the deformations of the vertebrae. Depending on the magnitude of deformation, corrections to the algorithm of spine reconstruction have revealed to be necessary [48].

Although the agreement between the radiological curve and the reconstructed curve is sufficient [49], the prediction of the radiological Cobb angle exhibits marked deviations from the radiological measure. Therefore, the prediction of the Cobb angle is often considered to be insufficient for clinical use. This may be due to several causes. Probably one mechanism blurring the prediction is caused by small local variations in the curve of surface rotation. In the reconstruction (cf. Figure 11), the distance L acts as a lever arm magnifying these small variations, which are superimposed to the reconstruction of the spinal midline. While these fluctuations have nearly no effect on the conformity of the spinal midline with the X-ray curve, they do adversely affect the determination of the Cobb angle taken from the tangents [50]. This is consistent with the observation that the level of the apical vertebra and the lateral deviation can be determined with satisfying precision.

Another component – however often enough ignored – is the reliability of the radiological Cobb angle itself being taken unjustified as a golden standard. It is compromised due to different sources of error [51]. The major errors arise from a residual variability in positioning the patient and an imprecise reading of the directions of the endplates [19].

## Orthopaedic relevance

**Figure 12:**
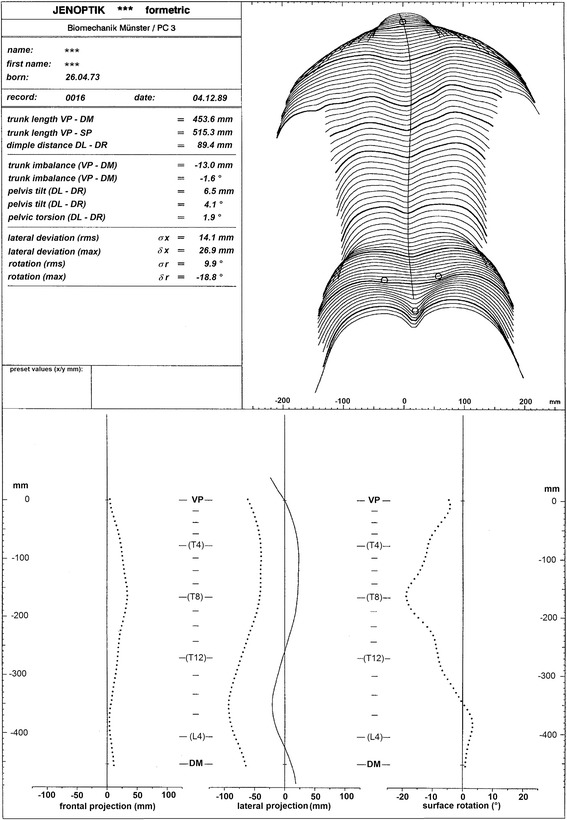
**Printout of shape analysis of a scoliotic back.**

An early version (1994) of a printout of a rasterstereographic reconstruction and analysis is given in Figure 12. The record was taken from a scoliotic patient at the age of 16 with a Cobb angle of 46°.

In the upper left field, the patient is identified, and a selection of back shape and spine shape parameters in the sagittal and frontal plane are given. Several parameters are indicated. All parameters – for mathematical reasons – either are independent of the patient’s position relative to the measuring system or refer to the line of gravity. However, independence of position must not be confused with independence of posture. Variations of posture may indeed affect parameters that are independent of position.

In particular, the parameters are as follows:

- “*trunk length*” is the spatial distance between the vertebra prominens landmark (VP) and either the midpoint (DM) between the two dimple landmarks (DL) and (DR) or the sacrum point (SP) landmark. Both definitions therefore are independent of the patient’s position relative to the imaging system.
- “*dimple distance*” indicates the spatial distance between DL and DR. This parameter is independent of the patient’s position relative to the imaging system too.
- “*trunk imbalance”* indicates the lateral deviation of VP from DM. A positive value means a shift of VP to the right.
- “*pelvis tilt*” refers to the difference in height of the lumbar dimples. A positive value means that the right dimple is higher than the left one.
- “*pelvic torsion*” describes the twisting of the pelvis about a transverse axis. It is calculated from the mutual twist of the surface normals at the lumbar dimples. Only the vertical component is considered. If the angle is positive, the right normal is pointing higher than the left one.
- “*lateral deviation (rms)*” indicates the root mean square deviation of the spinal midline from the line VP – DM in the frontal projection. See the lower part of the printout.
- “*lateral deviation (max)*” indicates the maximum deviation of the spinal midline from the line VP – DM in frontal projection. It is positive when on the right side.
- “*rotation (rms)*” indicates the root mean square deviation of the surface rotation. See the lower part of the printout. Without any spinal deformity, this value should be zero plus rms error of measurement.
- “*rotation (max)*” gives the maximum rms value. It is typically negative when the apex is on the right side.

In the upper right part of Figure 12, the back surface is represented by transverse profiles. The profiles are calculated along horizontal sections of the trunk, each separated by a vertical distance of 7.5 mm. The displayed profiles are in scale with the pertinent transverse surface profile, without a perspective effect. The type of representation exercised here permits exact measurements within one and the same profile. Vertical distances of points on different profiles cannot be measured directly but are determined by counting the profiles in between and taking into account their vertical distance of 7.5 mm each. In addition to the profiles, the symmetry line and anatomical landmarks are shown. The symmetry line passes – for reasons of symmetry – the VP landmark and runs in the middle between the left and right dimple landmark.

In the lower part of Figure 12, dotted lines show the frontal and lateral projection of the reconstruction of the spinal midline. The frontal projection on the left corresponds to an a.p. view of the spine. It reveals the side and the height of the apex. However, as has been pointed out above, reading the Cobb angle from tangents to the curve would be misleading. The lateral projection of the spinal midline is given in the middle. It provides, together with the frontal projection, a 3-D model of the spinal midline. The solid line beside it represents the lateral profile of the back, or more precisely, the lateral projection of the symmetry line. Both lateral projections reveal a reliable description of kyphotic and lordotic curvature of the vertebral body line and the surface. As has been pointed out, the curvature of the vertebral body line may differ from the radiological Cobb angle. The surface rotation ρ displayed on the right is defined by the horizontal deviation of the surface normals on the symmetry line from the sagittal plane. In a straight spine, the normals match the sagittal plane, and hence the rotation is expected to be zero. In interpreting this curve, the difference between the maximum angular deflection to the left and to the right also should be regarded. It too may be taken as a measure of the severity of the deformation.

On the left and right of the curves scales are given. Two of them are metric. Their origin is at the level of the VP landmark. The other two scales provide a proposition for the levels of T4, T8, T12 and L4 by interpolation between VP and DM. The approximative character of the proposition is underlined by the use of brackets in the labeling. In patients, for example, with the VP landmark indicating another tip than of C7 [36] this scale would have to be modified accordingly.

Regarding the usage of rasterstereography in a scoliosis clinic, both the max. and rms parameters of lateral deviation and surface rotation may be used to quantify progression, as has been shown recently [2]. The authors state that rasterstereography reflects the progression reliably and is comparable with the gold standard of radiography, particularly with regard to lateral vertebral deviation. The authors conclude that rasterstereography should be used as the technique of choice for recording the progression of scoliosis during the long-term follow-up, reducing the patient’s radiation exposure by approximately 50%.

## Discussion

Since its beginning [19], the usage of modern methods of optical back surface measurement techniques in the management of scoliosis has been dominated by the aspect of reducing radiation exposure due to radiological examinations. Radiological examinations are performed in diagnostic and follow-up examinations and carry an increased oncogenic risk. Regarding the general utility of these methods in a scoliosis clinic, reference is made to the Cobb angle as the golden standard. Reducing surface topography to the radiological Cobb angle reveals an ambivalent result: while it is evident that the prediction of the Cobb angle based on the back shape is not a fully adequate substitute for X-rays, it is observed that changes in back surface parallel changes in the Cobb angle and thus provide indications for radiology. Obtaining radiographs is advised only when a change in the surface topography is evident, indicating the need of an X-ray [2,3].

Furthermore, consensus exists regarding the accuracy and reproducibility of surface measurement techniques in general and of rasterstereography in particular. Therefore, surface topography is attested as feasible not only to follow patients with scoliosis but also to screen them [52].

It goes without saying that any information provided by back surface measurement that can be checked with X-rays should be checked with X-rays if available and that the closeness of agreement should provide a measure of reliability. As has been described here for rasterstereography, this comparison has been performed in scoliotic patients with deformities ranging from a light to severe condition exceeding 100° Cobb. The comparison in lateral and sagittal deviation and in surface rotation revealed results that were consistent and reliable, showing satisfactory agreement [2,42,53]. Investigations of other measurement systems and their clinical evaluation have been reviewed elsewhere [3].

However, stating this appreciation more or less from the aspect of the Cobb angle alone does not fully demonstrate the wealth of possibilities opened up by these new techniques. Obviously, there is information provided not by a standard frontal X-ray but in part only by the combination of a frontal and lateral X-ray. This extra information is exemplified by the true 3-D character of the information, the cosmetic conclusiveness and, finally, the absence of limitations in repeating measurements and thus the possibility of functional examinations [54,55] and diagnostic tests [53].

The wealth of information available for surface measurement needs to be used. In the case of rasterstereography, this is done using a sequence of different procedures incorporating image processing, mathematical shape analysis and biological modelling. Therefore, rasterstereography has been used here as a term to describe a method comprising the whole process from image capture to completion of evaluation with optimized and coordinated methods.

## Abbreviations

rms: Root mean square, a statistical measure
VP: Vertebra prominens landmark
DL/DR: Landmark attributed to the left/right dimple of Venus
SP: Sacrum point landmark
DM: Midpoint between left and right dimple landmark
